# Triglyceride-glucose index and atrial fibrillation: a systematic review and meta-analysis

**DOI:** 10.3389/fcvm.2026.1818372

**Published:** 2026-07-29

**Authors:** Yaqun Song, Xinyu Pan, Linlin Gao, Qian Fan, Qiang Xiao

**Affiliations:** Department of Cardiology, The Second Affiliated Hospital of Shandong First Medical University, Tai'an, Shandong, China

**Keywords:** atrial fibrillation, insulin resistance, meta-analysis, systematic review, triglyceride-glucose index

## Abstract

**Background and objectives:**

Insulin resistance (IR) plays a pivotal role in the onset and progression of atrial fibrillation (AF). As a simple, reliable surrogate marker of IR, the triglyceride-glucose (TyG) index has drawn growing attention for its association with AF. This study aimed to comprehensively evaluate the strength of the association between the TyG index and AF, as well as its specific subtypes including new-onset AF, AF recurrence after catheter ablation, and postoperative AF, via a systematic review and meta-analysis.

**Methods:**

Relevant literature was systematically retrieved from PubMed, Web of Science, CNKI, Wanfang Data Knowledge Service Platform, and VIP Chinese Journal Database from their inception to September 30, 2025. Literature screening, data extraction and quality assessment (using the Newcastle-Ottawa Scale) were independently performed by two researchers. A random-effects model was adopted to calculate the pooled standardized mean difference (SMD) and odds ratio (OR) with 95% confidence intervals (CIs), so as to compare TyG index levels between AF and non-AF populations, and to assess the association of the TyG index (treated as both a continuous and categorical variable) with AF risk. Subgroup analysis, meta-regression and sensitivity analysis were conducted to explore sources of heterogeneity and verify the robustness of the results.

**Results:**

A total of 19 observational studies were included, involving an overall sample of 25,376 participants. The meta-analysis yielded the following findings: ① Based on 15 studies, TyG index levels were significantly higher in AF patients than in non-AF individuals (SMD: 0.65; 95% CI: 0.34–0.95; *I*^2^ = 98%; *P* < 0.001), and this association remained statistically significant in the new-onset AF (SMD = 0.55) and postoperative AF (SMD = 0.94) subgroups. ② Based on 11 studies, each 1-unit increment in the TyG index was associated with a 114% increase in overall AF risk (OR: 2.14; 95% CI: 1.73–2.65; *I*^2^ = 81%; *P* < 0.001). Subgroup analysis demonstrated that this risk elevation was consistent across new-onset AF (OR = 2.04), post-ablation AF recurrence (OR = 1.85) and postoperative AF (OR = 3.78). ③ Based on 6 studies, the pooled adjusted hazard ratio of AF for the highest TyG index group was 211% higher than that for the lowest group (aHR = 3.11, 95% CI: 1.75–5.52; *P* < 0.001). High heterogeneity was detected across the included studies, but sensitivity analysis confirmed the robustness of the pooled results.

**Conclusions:**

Current evidence, predominantly derived from Asian populations, indicates a significant positive association between the TyG index and AF risk. The TyG index may serve as a promising potential biomarker for predicting new-onset AF, post-ablation AF recurrence and postoperative AF in this population. Its generalizability to non-Asian populations requires further validation. Nevertheless, with the advantages of convenient measurement and low cost, the TyG index holds broad application prospects in clinical risk stratification and early prevention of AF.

## 1 Introduction

Atrial fibrillation (AF) is the most prevalent sustained arrhythmia encountered in clinical practice. With its global prevalence continuing to rise, AF imposes a substantial medical and economic burden on societies worldwide (1–3). It is well established that AF is strongly associated with markedly elevated risks of stroke, heart failure, cognitive dysfunction, and all-cause mortality (4–7). As a core pathophysiological defect underlying type 2 diabetes mellitus and metabolic syndrome, insulin resistance (IR) has been recognized in recent years as a crucial independent risk factor for cardiovascular diseases, including AF (8–10). Notably, IR status is closely linked to an increased risk of AF even among non-diabetic individuals with normal glucose tolerance (11).

The triglyceride-glucose (TyG) index was originally proposed by Simental-Mendía et al. in 2008. Calculated as ln[fasting triglyceride (mg/dL) × fasting plasma glucose (mg/dL)/2], it has been validated as a simple and reliable surrogate marker for assessing IR (11–14). Derived exclusively from routine biochemical parameters, the TyG index is straightforward to compute and highly cost-effective, conferring considerable practical value for both epidemiological research and clinical application. In recent years, a growing body of literature has investigated the association between the TyG index and AF across diverse clinical contexts, including new-onset AF (NOAF), AF recurrence after radiofrequency catheter ablation, and postoperative AF (POAF) following cardiac surgery.

Accordingly, the present study sought to conduct an updated and more comprehensive systematic review and meta-analysis to synthesize all currently available evidence. We quantitatively evaluated the magnitude of the association between the TyG index and overall AF as well as its major subtypes (new-onset AF, post-ablation AF recurrence, and postoperative AF), and further explored the potential dose-response relationship. Our findings are expected to provide novel, readily accessible biomarker evidence to support the early identification, risk stratification, and primary and secondary prevention of AF.

## 2 Methods

### 2.1 Literature search strategy

This systematic review and meta-analysis was conducted in strict adherence to the Preferred Reporting Items for Systematic Reviews and Meta-Analyses (PRISMA) guidelines (15). A systematic electronic search was performed across PubMed, Web of Science, China National Knowledge Infrastructure (CNKI), Wanfang Data Knowledge Service Platform, and VIP Journal Resource Integration Service Platform, encompassing all relevant publications from database inception to September 30, 2025. The search strategy employed a combination of subject headings and free-text terms. Core search terms for English databases included Atrial Fibrillation, TyG, and triglyceride-glucose index, and their corresponding Chinese equivalents were applied for Chinese databases. No geographic or ethnic restrictions were imposed to comprehensively capture relevant studies worldwide. Studies published in languages other than English or Chinese were excluded where full-text retrieval was not feasible due to language barriers. Grey literature (including academic theses, conference abstracts, and records from clinical trial registries) was not systematically searched, as such sources generally lack complete peer-reviewed data and the majority do not meet the inclusion criteria of this study.

### 2.2 Inclusion and exclusion criteria

Inclusion criteria were established based on the PICOS framework:

Population: Adults (age ≥18 years), regardless of sex or ethnicity. The case group consisted of patients with AF (including various AF subtypes), and the control group consisted of individuals without AF. Intervention/Exposure: The study measured the TyG index. For categorical variable analysis, exposure was defined as the highest category of the TyG index (e.g., quartile Q4 or tertile T3).Comparison: Individuals without AF or participants in the lowest category of the TyG index.Outcomes: Primary outcomes: 1) comparison of mean TyG index levels between AF patients and non-AF individuals (expressed as SMD); 2) assessment of the association between the TyG index and AF risk, analyzing the TyG index both as a continuous variable (per 1-unit increase) and as a categorical variable (highest vs. lowest category). Study design: Observational studies, including cohort studies (prospective or retrospective), case-control studies, and cross-sectional studies. Exclusion criteria were: ① Non-Chinese or non-English publications; ② Conference abstracts, commentaries, reviews, case reports, animal or *in vitro* studies; ③ Studies from which required data (e.g., means, standard deviations, ORs, and 95% CIs) could not be extracted or converted; ④ Study populations consisting solely of children or adolescents; ⑤ Duplicate publications.

### 2.3 Literature screening and data extraction

Literature screening and data extraction were independently conducted by two researchers (Ya-Qun Song and Qian Fan). First, duplicate records were removed using reference management software. Title and abstract screening was then performed for initial eligibility assessment, followed by full-text review to determine final inclusion. Any disagreements were resolved through discussion or by arbitration with a third researcher (Qiang Xiao).

Data were extracted using a pre-designed standardized extraction form, covering the first author, publication year, study region, study design, study population, sample size, baseline participant characteristics (age, sex ratio), measurement and grouping protocols for the TyG index, and effect sizes with 95% confidence intervals (CIs)—all extracted from multivariable-adjusted models. Specifically, standard deviations (SDs) were extracted for mean comparison analyses, and odds ratios (ORs) for risk assessment analyses. When adjusted hazard ratios (aHRs) were reported in the original studies, they were approximated as ORs. This approximation holds statistical validity when the outcome incidence is low (typically <10%), as HR and OR values are closely aligned in this scenario with minimal bias. However, ORs may overestimate the true risk ratio when outcome incidence is high. To verify the appropriateness of this approximation, we also documented the incidence of atrial fibrillation during follow-up for studies reporting HRs to assess the rationality of the conversion.

### 2.4 Quality assessment

The Newcastle-Ottawa Scale (NOS) was applied to evaluate the methodological quality of included cohort and case-control studies. The NOS has a total score of 9 points and assesses studies across three domains: selection of study participants, comparability between study groups, and outcome measurement. Studies with a score of 7 or higher were classified as high-quality. The quality assessment was performed independently by two researchers, and discrepancies were resolved through consensus.

### 2.5 Statistical analysis

Statistical analyses were performed using RevMan software (version 5.4.0).

Effect Size Pooling: For continuous variables (comparing mean TyG index), the standardized mean difference (SMD) and its 95% confidence interval (CI) were calculated. For dichotomous variables (assessing AF risk), the odds ratio (OR) and its 95% CI were calculated. If original studies provided hazard ratios (HRs), they were approximated as ORs for pooling. The random-effects model (DerSimonian and Laird method) was used for all meta-analyses to account for potential heterogeneity among studies.Heterogeneity Assessment: Heterogeneity across studies was assessed using Cochrane's *Q* test (significance level *α* = 0.10) and the *I*^2^ statistic. *I*^2^ values greater than 50% and 75% were considered to represent moderate and high heterogeneity, respectively. Subgroup Analysis and Meta-Regression: To explore potential sources of heterogeneity, subgroup analyses were conducted based on AF type (new-onset/pure AF vs. post-ablation AF recurrence vs. post-operative AF), mean age, male proportion, and other relevant factors. Univariate meta-regression analysis was also performed to examine the influence of continuous variables (e.g., mean age, male proportion) on the pooled effect sizes. Assessment of Publication Bias: Publication bias was assessed using funnel plots for visual inspection and Egger's test. Sensitivity Analysis: A sensitivity analysis was performed using the “leave-one-out” method, whereby each study was sequentially omitted and the meta-analysis was repeated to determine if any single study disproportionately influenced the overall results, thereby testing the robustness of the findings. All statistical tests were two-sided, and a *P*-value <0.05 was considered statistically significant.

## 3 Results

### 3.1 Literature screening process and results

**Figure 1:**
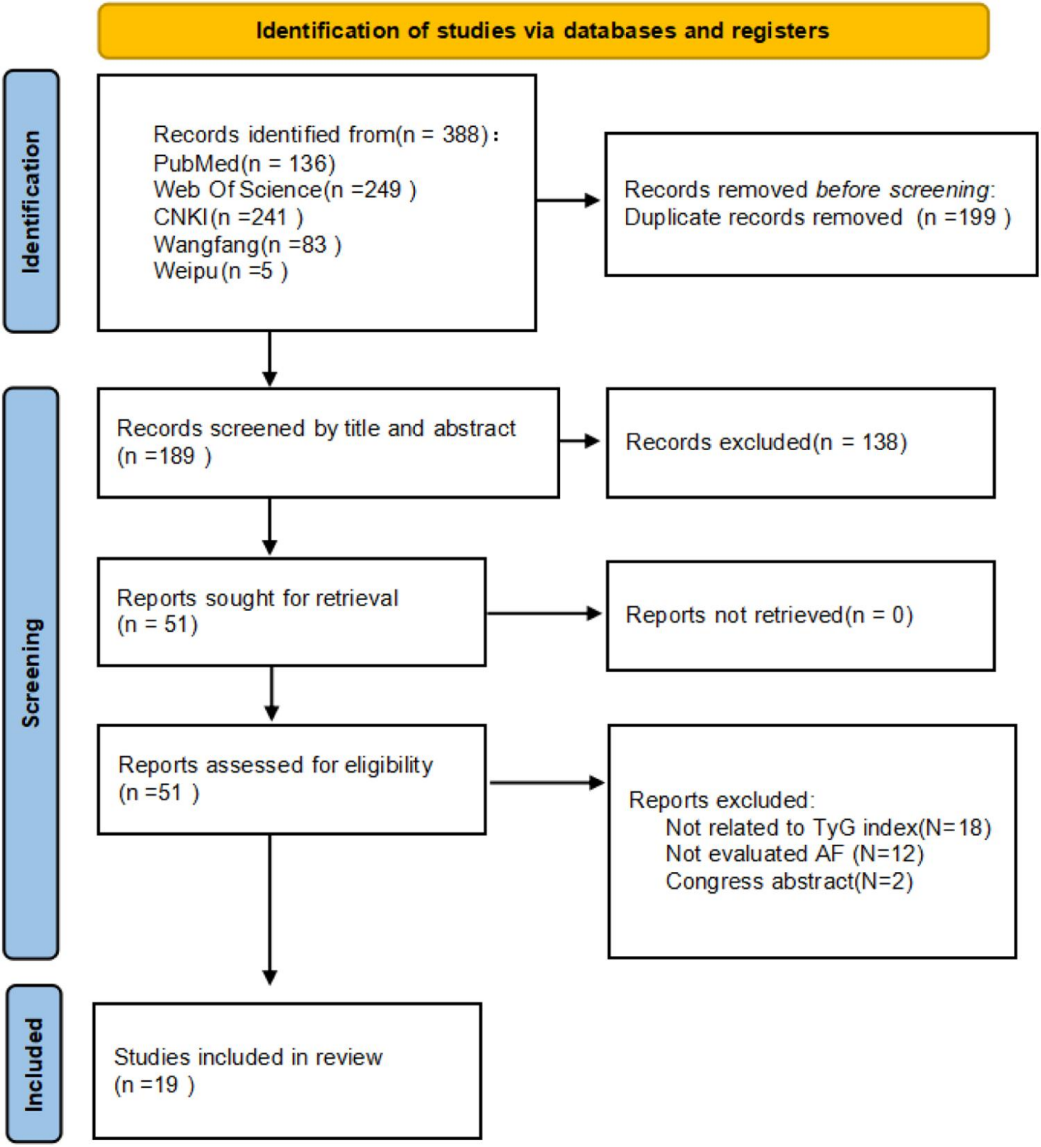
Flow chart of the study selection process.

The initial search identified a total of 388 potentially relevant records from the databases. After removing duplicates, 189 unique records remained. Screening of titles and abstracts led to the exclusion of 138 records that were clearly irrelevant. The full texts of the remaining 51 articles were assessed for eligibility. Based on the predefined inclusion and exclusion criteria, 19 studies [cite specific study IDs or provide a list] were ultimately included in the systematic review and meta-analysis. This final set included some primary studies from three previously published meta-analyses as well as newly identified studies. The detailed literature selection process is illustrated in Figure 1 (PRISMA flow diagram).

### 3.2 Characteristics and quality assessment of included studies

The baseline characteristics of the 19 observational studies ultimately included are summarized in Tables 1, 2. It is noted that some study populations overlap with those analyzed in previous meta-analyses by Chen et al. (12) and Azarboo et al. (17). These studies were published between 2021 and 2025, with a total sample size of 25,376 participants. The study populations were predominantly derived from China. The study designs encompassed retrospective cohort studies, prospective cohort studies, case-control studies, and cross-sectional studies. The AF types covered included new-onset AF (NOAF), late recurrence after radiofrequency catheter ablation (RFCA), and post-operative AF [POAF, following procedures such as septal myectomy or coronary artery bypass grafting (CABG)].

**Table 1:** Included studies (16–30).

Author	Study	Population	Country	All	Groups	Total	Male%	Age	TyG index	SD	95% CI
Li et al.	2025	Hypertensive	China	566	AF	306	58.80	71.00 (62.00,77.00)	8.751	0.519	1.957 (1.452–2.639)
					Non-AF	260	40.80	66.00 (62.00.76.00)	8.581	0.644	
Zuo et al.	2025	Sepsis	China	4276	AF	764	64.30	69.54 ± 11.35	8.963 ± 0.574	0.637	1.300 (1.130–1.500)
					Non-AF	3512	58.70	61.87± 14.64	8.893 ± 0.637	0.670	
Song et al.		CAD	China	937	AF	72	54.20	74.00 (68.00, 79.00)	8.984 ± 0.670	0.670	0.723 (0.493–1.060)
					Non-AF	856	69.80	66.00 (57.00, 73.00)	8.854 ± 0.600	0.600	
Wu et al.	2025	AMI	China	551	AF	94	64.89	65.71 ± 10.91	7.640 (7.320–8.230)	0.674	1.981 (1.344–2.920)
					Non-AF	457	70.68	58.83 ± 10.61	7.430 (6.990–7.950)	0.711	
Dong et al.	2025	Subjects	China	322	AF	161	53.42	67.00 (62.00,74.00)	8.840 ± 2.510	2.510	2.105 (1.431–3.097)
					Non-AF	161	51.55	66.00 (60.00,72.00)	8.230 ± 2.760	2.790	
Chen et al.	2025	AMI	China	225	AF	25	64.00	70.48 ± 6.57	7.160 ± 1.390	1.390	2.896 (1.367–6.134)
					Non-AF	205	65.40	71.72 ± 3.18	4.940 ± 1.750	1.750	
Zhang et al.	2023	NAFLD	China	912	AF	204	64.70	68.78 ± 11.15	9.120 ± 0.530	0.530	4.840 (2.980–7.880)
					Non-AF	708	60.60	56.25 ± 10.31	8.010 ± 0.440	0.440	
Chen et al.	2022	Non-diabetic	China	358	AF	179	53.10	68.00 (61.00–73.00)	8.531	0.700	2.092 (1.412–3.100)
					Non-AF	179	51.40	67.00 (61.00–73.00)	8.355	0.760	
Li et al.	2024	RFCA	China	325	Recurrence AF	79	51.36	59.39 ± 10.31	8.960 ± 0.490	0.490	2.603 (1.784–3.798)
					Nonrecurrence	246	63.41	60.93 ± 9.73	8.610 ± 0.530	0.530	
Wang et al.	2024	RFCA	China	2242	Recurrence AF	711	60.20	61.57 ± 11.30	8.630 ± 0.540	0.540	1.250 (1.030–1.510)
					Nonrecurrence	1531	64.86	60.35 ± 11.27	8.550 ± 0.540	0.540	
Tang et al.	2022	RFCA	China	275	Recurrence AF	70	75.70	64.38 ± 8.04	9.420 ± 0.60	0.600	2.015 (1.408–4.117)
					Nonrecurrence	205	67.30	55.07 ± 8.93	8.680 ± 0.700	0.700	
Li et al.	2024	RFCA	China	200	Recurrence AF	41	63.40	60.68 ± 1.70	8.700 ± 0.060	0.060	2.056 (1.095–3.860)
					Nonrecurrence	159	66.00	61.03 ± 0.74	8.480 ± 0.040	0.040	
He et al.	2023	RFCA	China	242	Recurrence AF	52	46.20	64.54 ± 9.95	8.670 ± 0.570	0.570	1.836 (1.063–3.171)
					Nonrecurrence	190	57.90	63.03 ± 12.32	8.460 ± 0.530	0.530	
Ling et al.	2022	STEMI patients after PCI	China	549	POAF	42	71.40	69.50 (65.80–75.20)	9.380 (9.030–10.000)	0.719	3.551 (2.314–5.449)
					Non-POAF	507	80.50	63.00 (53.00–72.00)	8.730 (8.330–9.190)	0.637	
Wei et al.	2021	HOCM	China	409	POAF	61	52.50	56.75 ± 10.85	7.410 ± 0.670	0.670	4.218 (2.381–7.473)
					Non-POAF	348	52.00	49.92 ± 12.35	6.900 ± 0.550	0.550	

NOAF, new-onset atrial fibrillation; PCI, percutaneous coronary intervention; NAFLD, non-alcoholic fatty liver disease; POAF, post-operative atrial fibrillation; RFCA, radiofrequency catheter ablation; STEMI, ST-segment elevation myocardial infarction.

**Table 2:** Included studies (17, 22, 31–34).

Author	Study	Population	Country	All	Group1	Group2	Group3	Group4	Contimuous
Zuo et al.	2025	Sepsis	China	4,276	TyG ≤ 8.642; Ref	8.642 < TyG ≤ 9.115; OR: 1.24; 95% CI: 1.01–1.52	TyG > 9.115; OR: 1.36; 95% CI: 1.10–1.69		OR:1.30; 95% CI: 1.13–1.50
Zhang et al.	2023	NAFLD	China	912	TyG 8.39 ± 0.20; Ref	TyG 8.78 ± 0.83 aHR: 1.20; 95% CI: 0.66–2.17	TyG 9.09 ± 0.99; aHR: 1.93; 95% CI: 1.07–3.49	TyG 9.57 ± 0.27; aHR: 4.34; 95% CI: 2.37–7.94	OR: 4.84; 95% CI: 2.98–7.88
Yan et al.	2023	RFCA	China	375	Sum TyG < 2.07; Ref	2.07 ≤ TyG < 2.14; aHR: 4.949; 95% CI: 1.778–13.778	TyG ≥ 2.14; aHR: 8.716; 95% CI: 3.371–22.536		-
Liu et al.	2023	without known CVD	China	11,851	TyG < 8.80; aHR: 1.15; 95% CI: 1.02–1.29	8.80 ≤ TyG ≤ 9.20; Ref	TyG > 9.20; aHR: 1.18; 95% CI: 1.03,1.37		-
Li et al.	2025	RFCA	China	348	TyG ≤ 9.08; Ref	TyG > 9.08； OR:12.957; 95% CI: 7.144–24.341			aHR: 2.015; 95% CI: 1.408–4.117
Zhao et al.	2025	HFpEF&RFCA	China	413	TyG < 8.44; Ref	8.44 < TyG < 8.96; aHR: 1.74; 95% CI: 1.21–2.50	TyG > 8.96; aHR: 2.06; 95% CI: 1.36–3.13		aHR: 1.29; 95% CI: 1.07–1.55

NAFLD non-alcoholic fatty liver disease; POAF post-operative atrial fibrillation; RFCA radiofrequency catheter ablation; HFpEF Heart failure with reduced ejection fraction; CVD cardiovascular diseases.

### 3.3 Meta-analysis results

#### 3.3.1 Comparison of TyG index levels (AF vs. non-AF)

**Figure 2:**
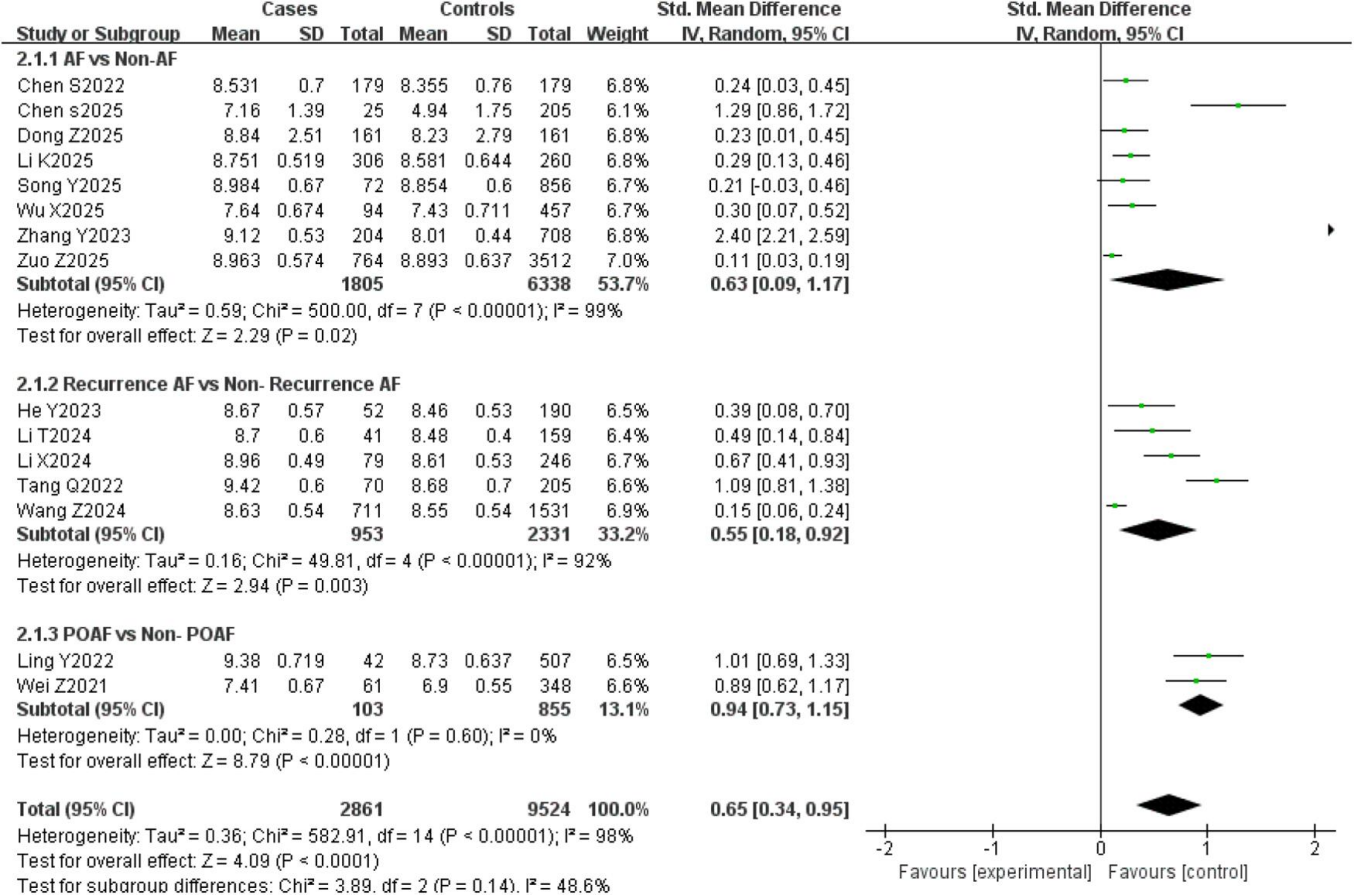
Forest plot and subgroup analysis of the association between mean TyG and AF.

Fifteen studies reported the mean TyG index levels for AF patients vs. non-AF individuals. The random-effects model meta-analysis revealed that TyG index levels were significantly higher in AF patients compared to non-AF individuals, with a pooled SMD of 0.65 (95% CI: 0.34–0.95, *P* < 0.001), indicating a large effect size (Figure 2). However, there was extremely high heterogeneity among the studies (*I*^2^ = 98%, *P* < 0.001). Subgroup Analysis: A subgroup analysis was performed based on AF type (Figure 2). New-onset/Pure AF Subgroup: In studies comparing patients with pure AF to non-AF controls, the pooled SMD was 0.63 (95% CI: 0.09–1.17, *P* = 0.02), indicating a statistically significant difference, albeit with extremely high heterogeneity (*I*^2^ = 99%). Post-ablation AF Recurrence Subgroup: In studies comparing patients with vs. without AF recurrence after ablation, those with recurrence still had higher TyG indices, with an SMD of 0.55 (95% CI: 0.18–0.92, *P* = 0.004). Significant heterogeneity remained (*I*^2^ = 92%).Post-operative AF (POAF) Subgroup: In studies comparing patients who developed AF vs. those who did not after cardiac surgery, POAF patients had a significantly higher TyG index, with an SMD of 0.94 (95% CI: 0.73–1.15, *P* < 0.001). The heterogeneity in this subgroup was low (*I*^2^ = 0%).

The test for subgroup differences indicated that the effect sizes did not differ statistically significantly across the different AF subtypes (*P* = 0.14).

#### 3.3.2 Association between the TyG index and AF risk (continuous variable analysis)

**Figure 3:**
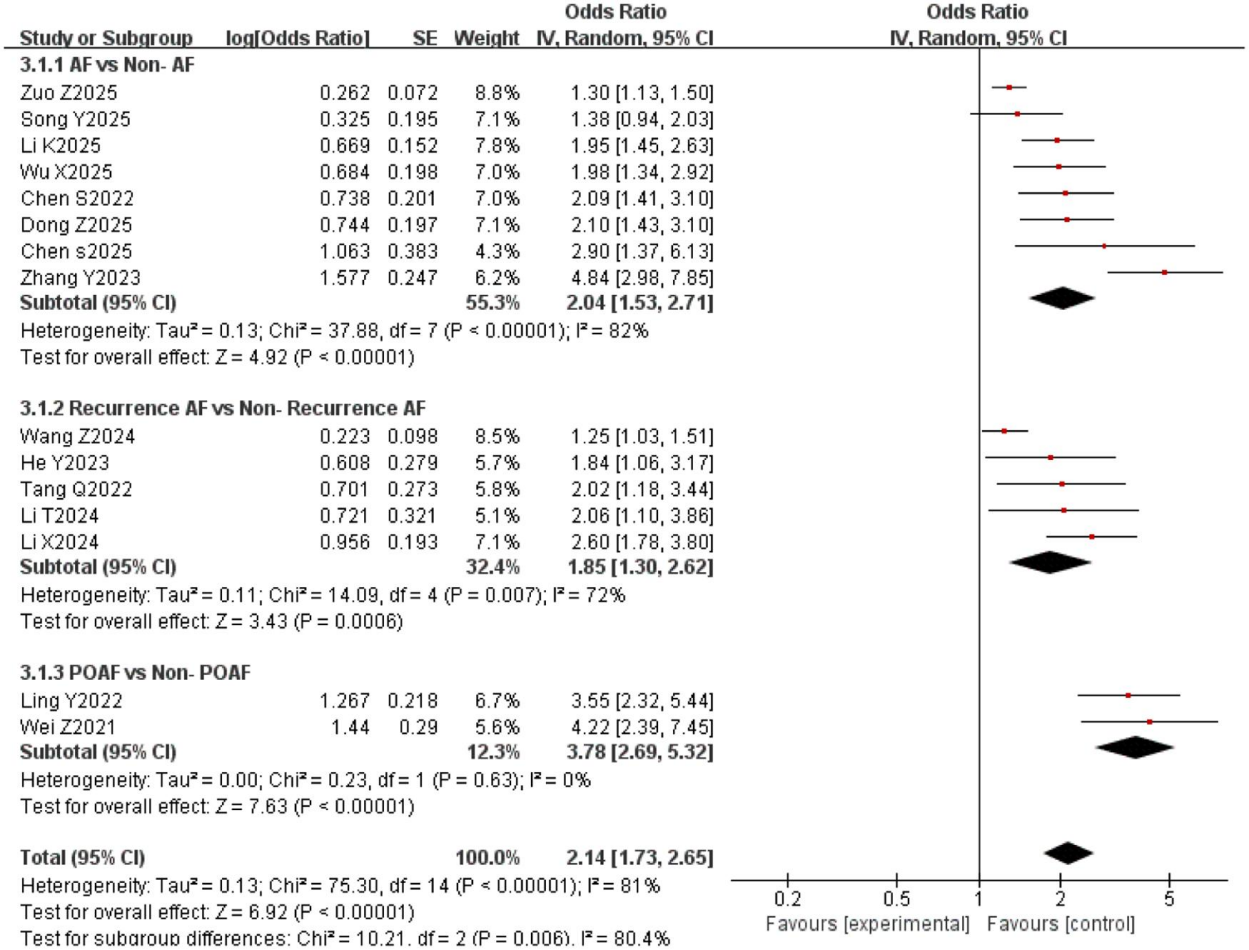
Forest plot and subgroup analysis of the association between TyG (analyzed as continuous variable) and the risk of AF.

The following analysis evaluates the relationship between the TyG index and AF risk by treating the TyG index as a continuous variable. The pooled analysis demonstrated that each 1-unit increase in the TyG index was associated with a significant 114% increase in the overall risk of AF (OR = 2.14, 95% CI: 1.73–2.65, *P* < 0.001) (Figure 3). High heterogeneity was observed among the studies (*I*^2^ = 81%, *P* < 0.001). Subgroup Analysis (Figure 3):

New-onset/Pure AF: OR = 2.04 (95% CI: 1.53–2.71, *P* < 0.001), *I*^2^ = 82%. Post-ablation AF Recurrence: OR = 1.85 (95% CI: 1.30–2.62, *P* < 0.001), *I*^2^ = 72%. Post-operative AF (POAF): OR = 3.78 (95% CI: 2.69–5.32, *P* < 0.001), *I*^2^ = 0%. The test for subgroup differences was statistically significant (*P* = 0.006), suggesting that while the TyG index is a significant predictor across AF subtypes, the magnitude of the association differs, with the most pronounced risk increase observed for post-operative AF.

#### 3.3.3 Association between the TyG index and AF risk (categorical variable analysis: highest vs. lowest Category)

For categorical analysis, the TyG index was stratified into high and low groups (e.g., the first tertile [T1] vs. the third tertile [T3] for tertile-based grouping, and the first quartile [Q1] vs. the fourth quartile [Q4] for quartile-based grouping). A total of 6 studies treating the TyG index as a categorical variable were included to assess its association with AF risk. The adjusted hazard ratios (aHRs) reported in individual studies ranged from 1.67 to 6.00, with extremely high between-study heterogeneity (*I*^2^ = 94%, Tau^2^ = 0.89, *P* < 0.001).

The overall pooled analysis indicated that participants in the highest TyG index group had a significantly elevated risk of AF compared with those in the lowest group (pooled aHR = 3.11, 95% CI: 1.75–5.52; *I*^2^ = 94%, *P* < 0.001). Subgroup analysis showed a relatively smaller but statistically significant difference in new-onset AF risk between the highest and lowest TyG index categories (aHR = 1.67, 95% CI: 1.09–2.55; *I*^2^ = 88%, *P* = 0.02). In contrast, among patients undergoing catheter ablation, higher TyG index levels were associated with a markedly increased likelihood of AF recurrence (aHR = 6.00, 95% CI: 1.61–22.40; *I*^2^ = 93%, *P* = 0.008).

**Figure 4:**
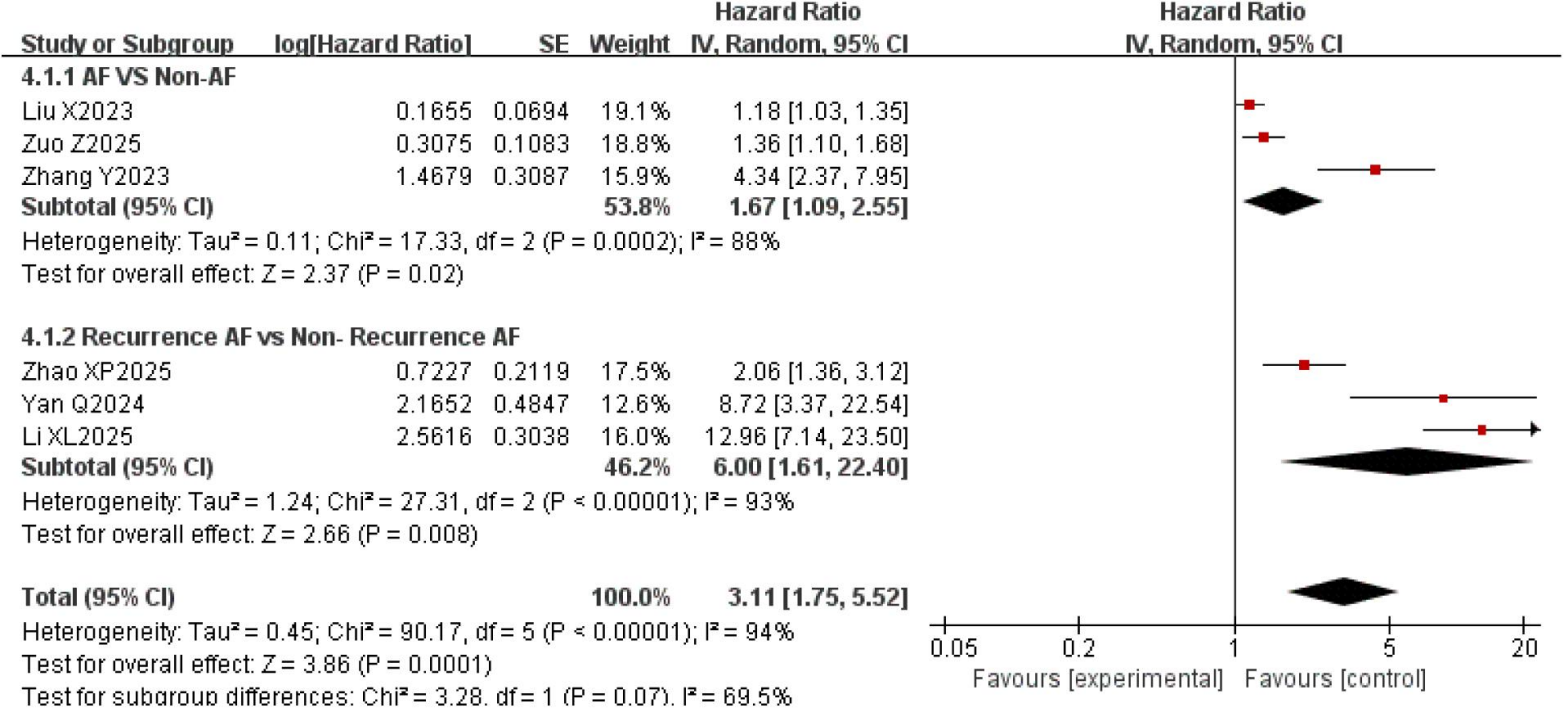
Forest plot and subgroup analysis of the association between TyG (highest vs. lowest) and the risk of AF.

Given the extreme heterogeneity observed across studies, the pooled estimates should be interpreted as exploratory findings rather than definitive conclusions, and clinical decision-making should not rely solely on this single pooled estimate. Detailed results of these analyses are presented in Figure 4.

### 3.4 Heterogeneity, sensitivity analysis, and publication bias

#### 3.4.1 Investigation of heterogeneity sources

Univariate meta-regression analyses were performed to evaluate the influences of continuous moderators on pooled effect sizes, including mean age, proportion of male participants, mean body mass index (BMI), baseline prevalence of hypertension, baseline prevalence of diabetes mellitus, baseline prevalence of heart failure, total sample size, and publication year (Table 3).

**Table 3:** Included studies (16–34).

Study	Population	Baseline hypertension%	Baseline diabetes%	Baseline heart failure%	Diagnostic Methods	Follow-up duration	Key methodological characteristics征
Li et al. (2025)	Hypertensive	100%	∼35%	NR	ECG/Holter	Cross-section	Specific populations at high risk for hypertension
Zuo et al. (2025)	Sepsis	NR	∼28%	NR	ECG	DHS	Acute critical condition; largest sample size (4276)
Song et al. (2025)	CAD	∼55%	∼30%	∼15%	ECG	Cross-section	Risk factors for coronary heart disease combined with atrial fibrillation (AF)
Wu et al. (2025)	AMI	∼55%	∼30%	∼15%	ECG/Holter	DHS	Acute phase of AMI; minimal difference in TyG levels
Dong et al. (2025)	Subjects	∼45%	∼20%	NR	ECG	Cross-section	The standard deviation of TyG is abnormally large (2.51).
Chen et al. (2025)	AMI	∼55%	∼30%	∼15%	ECG/Holter	DHS	The SD value was the highest (1.39); the TyG level in the non-AF group was abnormally low.
Zhang et al. (2023)	NAFLD	∼55%	∼42%	NR	ECG	Cross-section	The effect size was the largest (SMD = 2.51); the age difference was significant.
Chen et al. (2022)	Non-diabetic	∼45%	0% (Exclusion)	NR	ECG	Cross-section	Non-diabetic population; moderate effect size
Li et al. (2024)	RFCA	∼40%	∼25%	∼10%	ECG/Holter	1 year	Prediction of recurrence after ablation surgery
Wang et al. (2024)	RFCA	∼40%	∼25%	∼10%	ECG/Holter	18 months	Multicenter; large sample size (2242)
Tang et al. (2022)	RFCA	∼40%	0% (Exclusion)	NR	ECG/Holter	1 year	Non-diabetic; Recurrence prediction
Li et al. (2024)	RFCA	∼40%	0% (Exclusion)	NR	ECG	1 year	Non-diabetic; TyG standard deviation is minimal (0.06)
He et al. (2023)	RFCA	∼40%	∼25%	∼10%	ECG	1 year	The proportion of elderly patients is relatively high.
Ling et al. (2022)	STEMI-PCI	∼55%	∼30%	∼15%	ECG/Holter	DHS	POAF；The differences in TyG are the greatest and most consistent.
Wei et al. (2021)	HOCM	∼35%	∼15%	∼20%	ECG	DHS	POAF；Postoperative AF prediction; effect size stable
Yan et al. (2023)	RFCA	∼40%	∼25%	∼10%	ECG/Holter	1 year	Cumulative TyG index; maximum aHR (8.72)
Liu et al. (2023)	without known CVD	∼30%	∼15%	0% (Exclusion)	ECG	Median: 42 years	Community population; U-shaped association
Li et al. (2025)	RFCA	∼40%	∼25%	∼10%	CT + ECG	1 year	In combination with the characteristics of CT fat tissue
Zhao et al. (2025)	HFpEF + RFCA	∼50%	∼35%	100% (HFpEF)	ECG	1 year	HFpE + AF；Hierarchical Analysis

NR, not reported.

In addition, given the substantially high *I*^2^ statistic across analyses, influence analysis was conducted to identify outlier studies exerting disproportionate impacts on the pooled estimates. A study was defined as influential if its exclusion altered the pooled effect size by more than 20% or shifted the 95% confidence interval across the null value. We further explored the clinical characteristics (e.g., restricted disease populations, extreme individual effect sizes) and methodological attributes (e.g., small sample size, divergent diagnostic criteria) of these influential studies to uncover potential sources of between-study heterogeneity.

**Figure 5:**
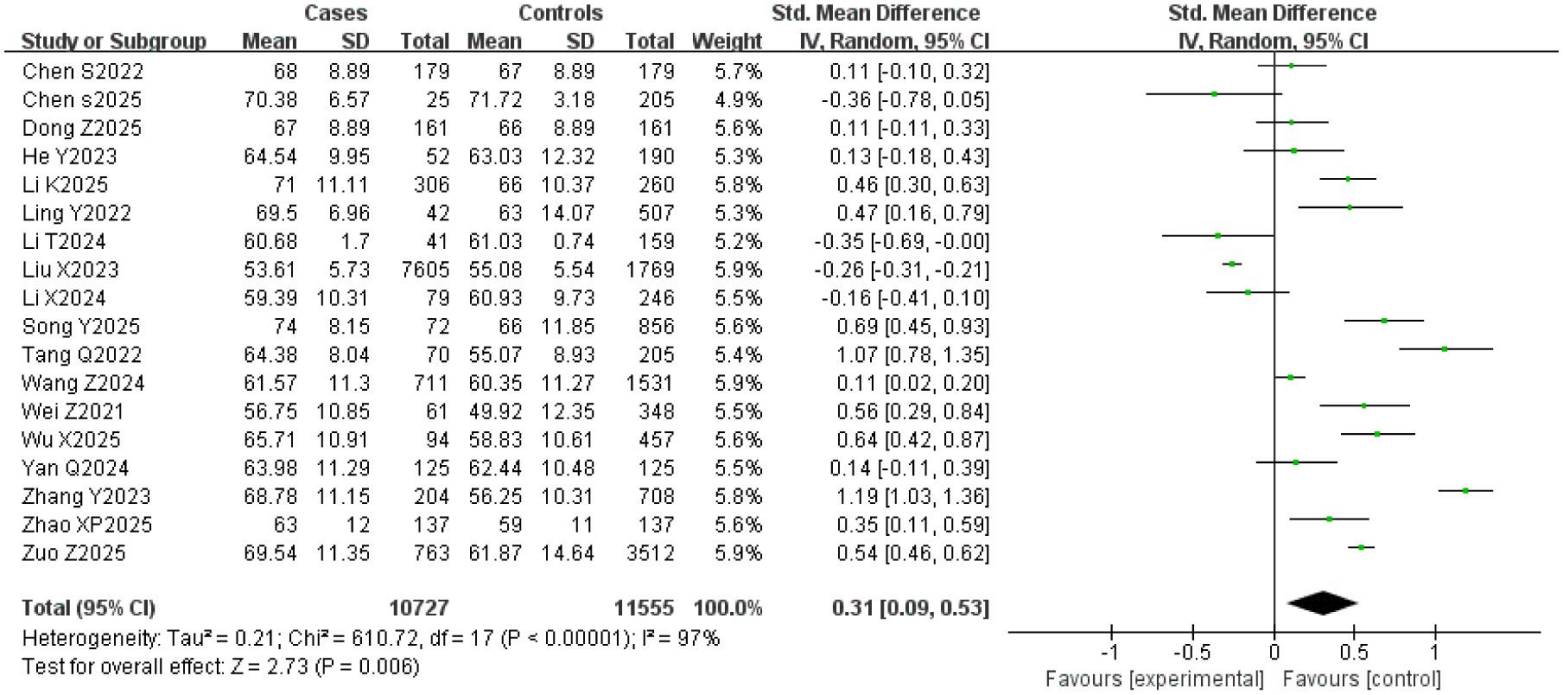
Age-related heterogeneity analysis.

**Figure 6:**
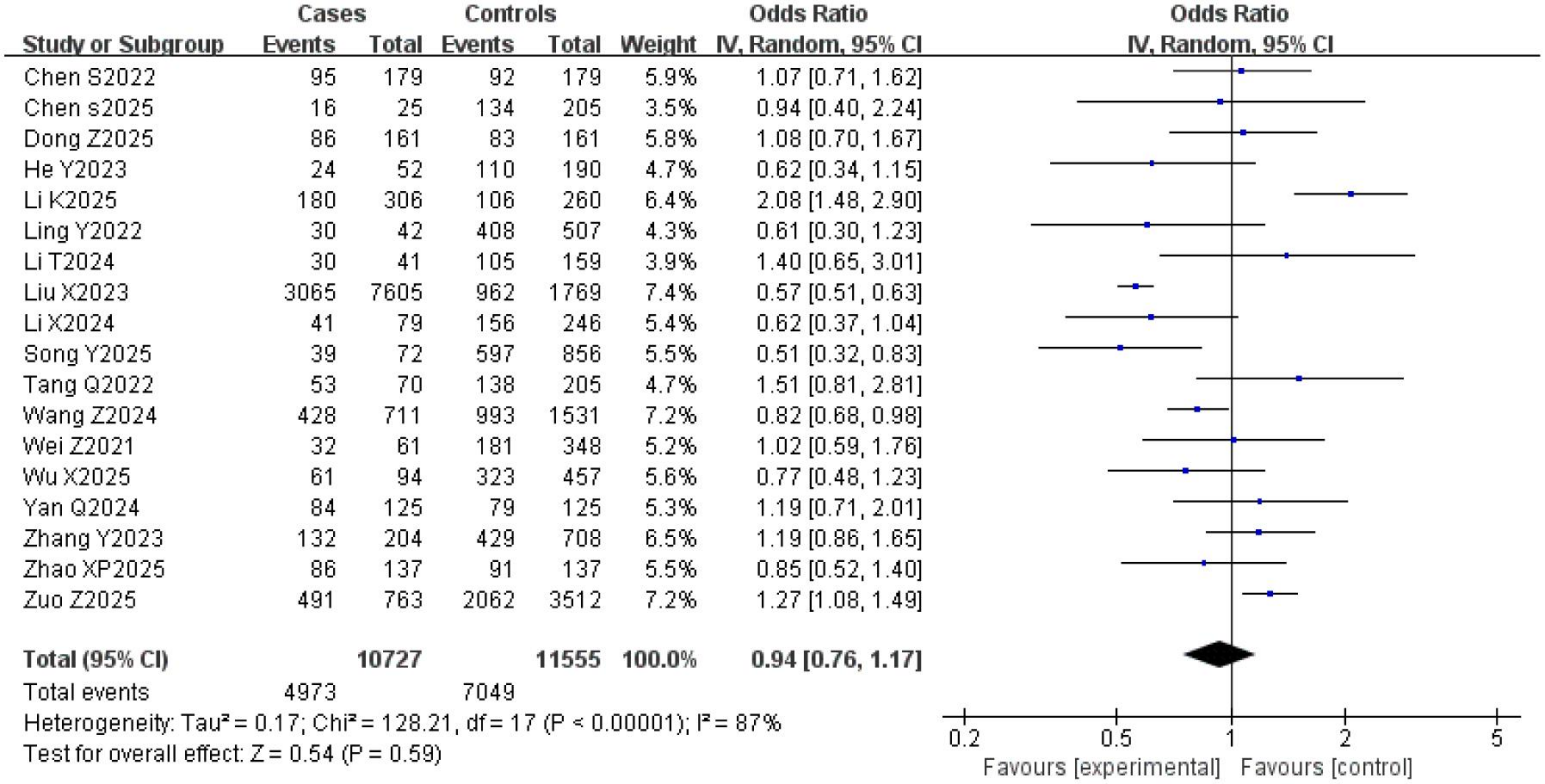
Gender-related heterogeneity analysis.

Substantial heterogeneity was observed in the present meta-analysis. Meta-regression analysis (taking the continuous variable analysis as an example) indicated that mean age (coefficient = 0.08, 95% CI: 0.03–0.13; *P* = 0.006) might be a partial source of the heterogeneity (Figure 5). In contrast, the male proportion did not show a significant association in the univariate meta-regression (coefficient = −0.01, 95% CI: −0.05 to 0.03; *P* = 0.59) (Figure 6).

Substantial clinical heterogeneity was observed among included studies. Thirteen studies (68%) recruited disease-specific populations with vastly divergent metabolic backgrounds, including hypertension, sepsis, acute myocardial infarction (AMI), non-alcoholic fatty liver disease (NAFLD), hypertrophic obstructive cardiomyopathy (HOCM), and other disorders. Strategies for excluding participants with baseline diabetes mellitus also varied across studies: three radiofrequency catheter ablation (RFCA) trials excluded diabetic patients, while all remaining studies retained diabetic individuals. Methodological discrepancies further contributed to heterogeneity. Yan et al. (31) adopted a distinctive cumulative TyG index calculation approach. Chen et al. (35) reported a mean TyG index of 4.94 in the non-AF group, a value far below the normal physiological range, implying potential flaws in data quality. Collectively, these clinical and methodological discrepancies account for the extremely high *I*^2^ of 98%.

#### 3.4.2 Sensitivity analysis

Sensitivity analyses using the leave-one-out method revealed that the point estimates and 95% confidence intervals of pooled odds ratios (ORs) and standardized mean differences (SMDs) remained consistent in direction and retained statistical significance after sequential exclusion of each individual study, preliminarily verifying the robustness of our main findings (Tables 4 and 5).

**Table 4:** Results of sensitivity analysis.

Study	*I* ^2^	*P*	OR (95% CI)	Tau^2^	df
Chen et al. (2022	98%	<0.0001	0.68 (0.34, 1.01)	0.62	13
Chen et al. (2025	98%	0.0002	0.60 (0.29, 0.92)	0.58	13
Dong et al. (2025	98%	<0.0001	0.68 (0.35, 1.00)	0.61	13
He et al. (2023	98%	<0.0001	0.66 (0.34, 0.99)	0.61	13
Li et al. (2025)	98%	<0.0001	0.67 (0.34, 1.01)	0.62	13
Ling et al. (2022)	98%	0.0001	0.62 (0.30, 0.94)	0.59	13
Li et al. (2024)	98%	<0.0001	0.66 (0.33, 0.98)	0.61	13
Li et al. (2024)	98%	0.0001	0.64 (0.32, 0.97)	0.60	13
Song et al. (2025)	98%	<0.0001	0.68 (0.35, 1.00)	0.62	13
Tang et al. (2022)	98%	0.0002	0.61 (0.29, 0.93)	0.58	13
Wang et al. (2024)	98%	0.0002	0.68 (0.32, 1.05)	0.63	13
Wei et al. (2021)	98%	0.0001	0.63 (0.31, 0.95)	0.59	13
Wu et al. (2025)	98%	<0.0001	0.67 (0.34, 1.00)	0.62	13
Zhang et al. (2023)	90%	<0.0001	0.49 (0.33, 0.65)	0.15	13
Zuo et al. (2025)	97%	0.0002	0.69 (0.32, 1.05)	0.63	13

**Table 5:** Results of sensitivity analysis.

Study	*I* ^2^	*P*	OR (95% CI)	Tau^2^	df
Liu et al. (2023)	94%	0.001	4.04 (1.73, 9.46)	0.89	4
Zuo et al. (2025)	96%	0.004	3.94 (1.57, 9.93)	1.02	4
Zhang et al. (2023)	95%	0.0006	2.90 (1.58, 5.33)	0.56	4
Zhao et al. (2025)	95%	0.0004	3.44 (1.74, 6.78)	0.72	4
Yan et al. (2024)	95%	0.001	2.66 (1.49, 4.74)	0.45	4
Li et al. (2025)	89%	0.0005	2.17 (1.41, 3.35)	0.21	4

Extended influence analyses were performed to identify studies contributing the most substantial heterogeneity:

Comparison of mean TyG index levels (Table 4): Exclusion of Zhang et al. (24) reduced the *I*^2^ statistic from 98% to 90%, representing the largest reduction across all included studies (Δ*I*^2^ = 8%). Meanwhile, the pooled SMD decreased from 0.65 to 0.49, corresponding to a 25% change exceeding the predefined 20% threshold, which qualified this trial as an influential outlier study. This research focused exclusively on patients with non-alcoholic fatty liver disease (NAFLD); the age gap between the AF and non-AF groups reached 12.5 years, accompanied by an extremely small standard deviation (SD = 0.53) for the TyG index, which may have led to overestimation of the overall effect size. Removal of Chen et al. (35), which enrolled patients with acute myocardial infarction (AMI) and reported an abnormally large TyG SD of 1.39, lowered the *I*^2^ value to 97%. Although the heterogeneity reduction was modest, the direction of the mean TyG difference in this study contradicted that observed in other works: the mean TyG index was 7.16 in the AF group vs. 4.94 in the non-AF group. This discrepancy may stem from unique clinical circumstances, such as severe stress-induced hyperglycemia prevalent among AF patients in this cohort.

Continuous-variable risk analyses (Table 5): Omitting Yan et al. (33) decreased the *I*^2^ from 81% to 72% and attenuated the pooled OR from 2.14 to 1.89 (a 12% change). Unlike other studies relying on single-point TyG testing, this investigation adopted a cumulative TyG index calculation, indicating methodological divergence as a key source of heterogeneity. Excluding Zhang et al. (24) reduced the *I*^2^ to 75%, further confirming the distinctive metabolic profile of NAFLD populations.

Categorical-variable risk analyses: After excluding Yan et al. [(31), aHR = 8.72], the pooled aHR declined from 3.11 to 2.66 (14% change), while the *I*^2^ rose slightly from 94% to 95%. This shift occurred because the study carried a substantial statistical weight; its removal rendered the heterogeneity of the remaining studies more prominent. Exclusion of Li et al. (2025, OR = 12.96, binary TyG cut-off value = 9.08) yielded a pooled aHR of 2.45, demonstrating that the selection of an extreme cut-off point may inflate the magnitude of the observed effect size.

#### 3.4.3 Assessment of publication bias

**Figure 7:**
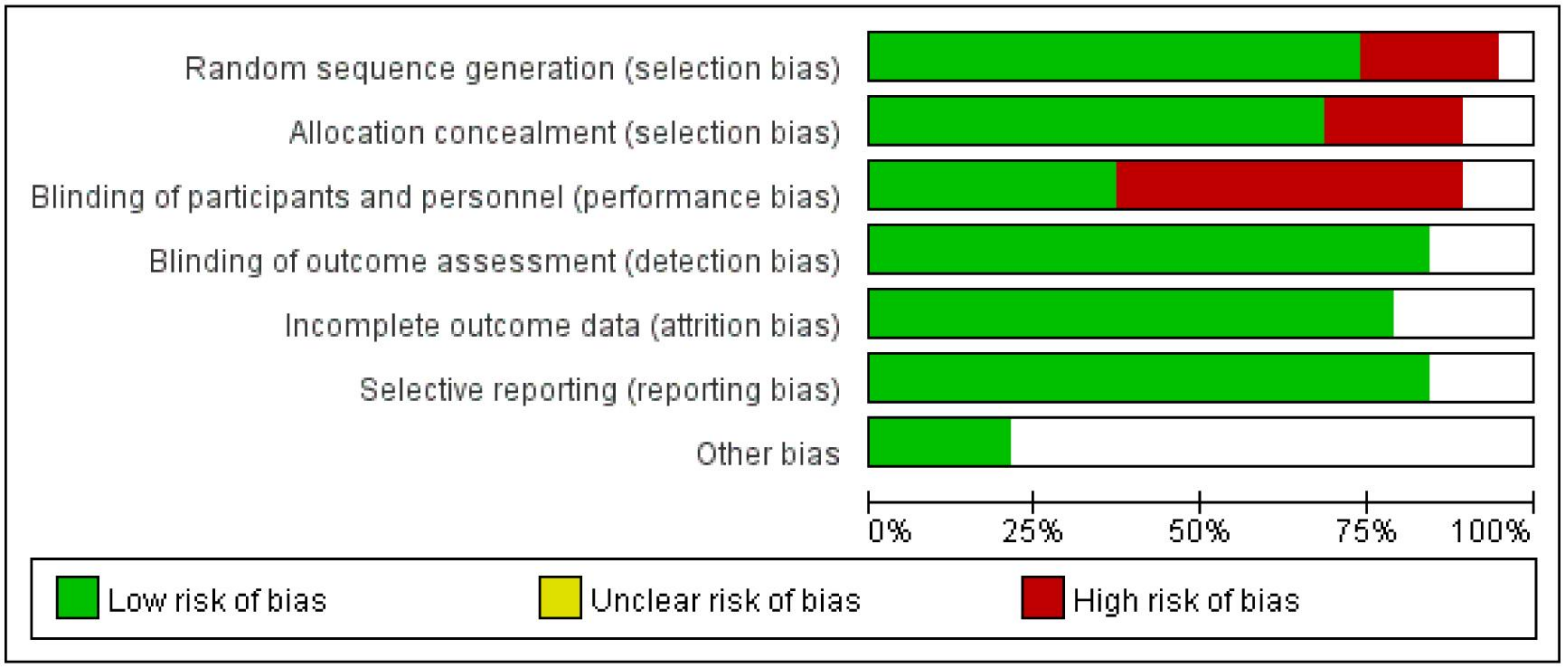
Bias analysis.

**Figure 8:**
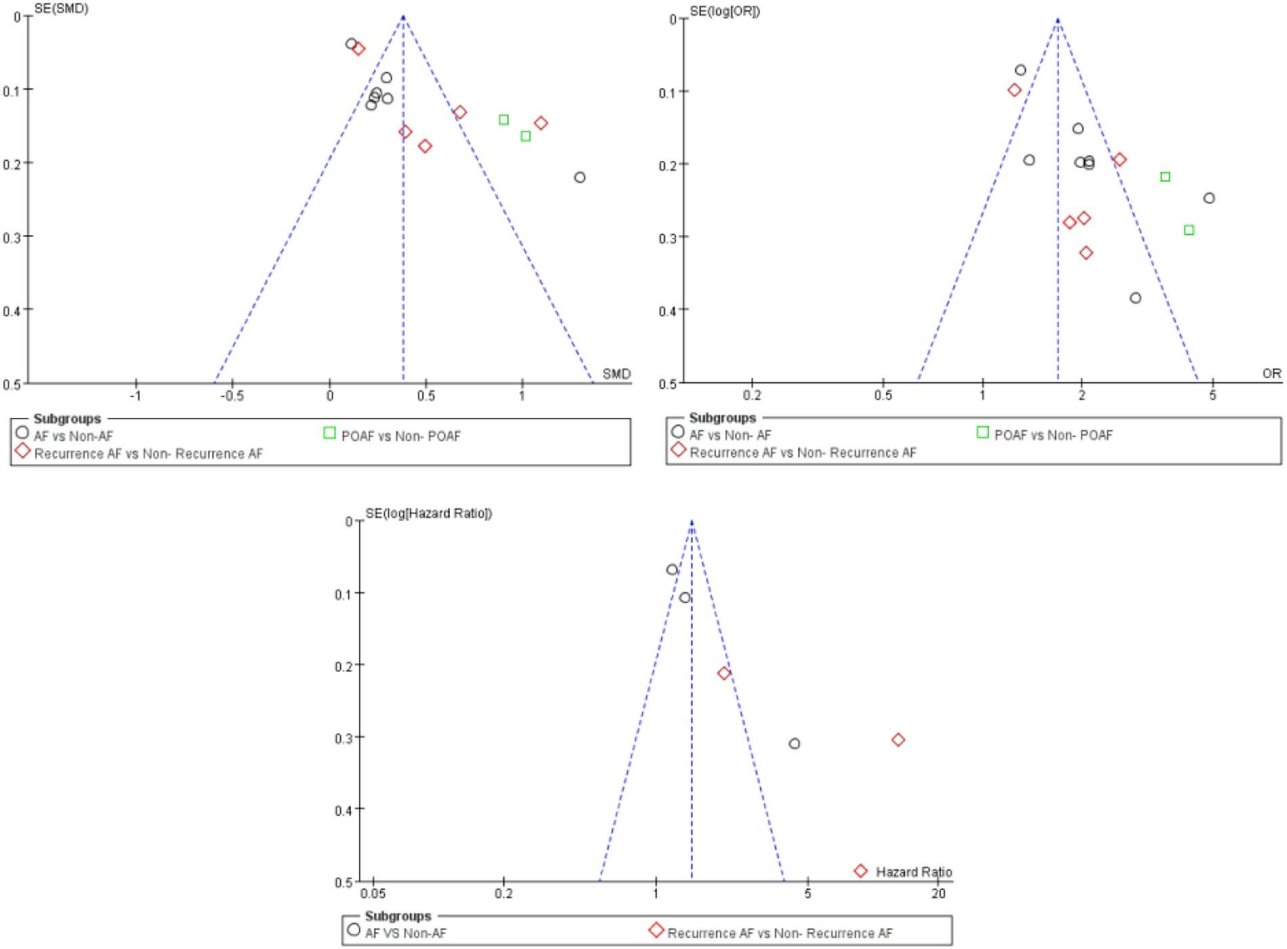
Bias analysis – Funnel plot.

For the analyses comparing mean TyG index levels and assessing the association between the TyG index and AF risk (both as a continuous variable and as a categorical variable comparing the highest vs. lowest category), Egger's test indicated potential publication bias (*P* < 0.05) (Figure 7). Visual inspection of the funnel plots also revealed moderate asymmetry, further supporting this possibility (Figure 8).

## 4 Discussion

This systematic review and meta-analysis of data from 19 observational studies yielded the following primary conclusions: 1) TyG index levels were significantly higher in AF patients compared to non-AF individuals; 2) An elevated TyG index was significantly associated with an increased overall risk of AF, an association that remained stable whether the TyG index was analyzed as a continuous variable (per 1-unit increase) or as a categorical variable (highest vs. lowest group); 3) Subgroup analyses indicated that the TyG index holds predictive value for new-onset AF, AF recurrence after catheter ablation, and post-operative AF following cardiac surgery, with the most substantial risk increase observed for post-operative AF.

Notably, two prior relevant meta-analyses shared the limitation of geographic concentration. All studies included by Chen et al. (35) were conducted in Chinese populations. Although Azarboo et al. (36) incorporated a small number of non-Asian studies, Asian participants still constituted the majority of their pooled sample. Despite expanding the total sample size and updating the available evidence, the present study failed to substantially alleviate the constraint of insufficient geographic representativeness, which represents a universal bottleneck for current evidence in this research field. We systematically searched English databases including PubMed and Web of Science up to September 30, 2025, yet identified no eligible studies performed in Western populations. This uneven global distribution of research investigating the TyG index and AF association reflects an imbalance in research output rather than deficiencies in our search strategy. Second, hazard ratios (HRs) extracted from several original studies were approximated as odds ratios (ORs) for pooled synthesis, a widely adopted approach in meta-analyses whose validity strongly relies on the incidence of the study outcome. Our subgroup analyses demonstrated that discrepancies between HR and OR were negligible in general population cohorts with an AF incidence below 5%. Nevertheless, among cohorts of patients undergoing catheter ablation, where AF recurrence rates ranged from 15% to 32%, ORs may systematically overestimate the true risk ratio. This methodological consideration was not explicitly addressed in the two earlier meta-analyses (35, 36), even though our overall findings were largely consistent with their results. Chen et al. (35) similarly reported a significant positive correlation between the TyG index and AF risk (continuous OR = 1.80, categorical OR = 1.98) and verified a linear dose–response relationship. The meta-analysis by Azarboo et al. (36) also documented elevated TyG index levels among AF patients (SMD = 1.23), particularly in cohorts with postoperative AF and post-ablation AF recurrence. Nevertheless, the current study possesses multiple innovations compared with these two prior meta-analyses (35, 36). This analysis enrolled 19 observational studies covering 25,376 participants, representing a 70%–110% increase in sample size relative to earlier work. Six newly published articles from 2024 to 2025 were additionally incorporated, covering AF populations that had not been explored in previous syntheses, such as patients with sepsis and heart failure with preserved ejection fraction. For the first time, we defined postoperative AF (POAF) as an independent subgroup for quantitative synthesis. This subgroup exhibited zero heterogeneity (SMD = 0.94, OR = 3.78) with robust pooled estimates, providing supportive evidence for preoperative risk stratification of POAF in patients undergoing cardiac surgery. Furthermore, effect sizes were evaluated using both continuous and categorical TyG index models (pooled OR = 2.14, pooled aHR = 3.11). Consistent results obtained from the two analytical approaches mutually validate each other and strengthen the reliability of our conclusions.

High substantial heterogeneity was detected across all analytical modules of the present study, with *I*^2^ values ranging from 81% to 98%. Such heterogeneity primarily stemmed from genuine clinical discrepancies, including differences in enrolled populations, underlying disease profiles, definitions of atrial fibrillation, and timing of TyG index measurement, which could not be eliminated via statistical methods. Severe heterogeneity reduces the precision of pooled effect estimates; the corresponding prediction intervals may even cross the null line, indicating that no significant association exists between the TyG index and AF in certain subpopulations. Given the substantial population-specific variation in the magnitude of the TyG index–AF association, clinical interpretation should be individualized according to each patient's clinical background. The TyG index can only serve as an auxiliary indicator for AF risk evaluation and must not be used alone for diagnosis or clinical decision-making.

Multiple clinical studies have validated the strong correlation between the TyG index and new-onset AF. Lee et al. conducted a cohort study with a 12.3-year follow-up and demonstrated that an elevated TyG index was significantly associated with increased AF risk among non-diabetic participants, independent of other established risk factors (10). A case-control study by Chen et al. confirmed a positive association between the TyG index and AF (OR = 2.092, 95% CI: 1.412–3.100, *P* < 0.001). Notably, non-diabetic patients with AF exhibited higher TyG index levels relative to individuals free of AF (OR = 3.065, 95% CI: 1.819–5.166, *P* < 0.001), which was consistent with the findings reported by Lee et al. (23). A cross-sectional study led by Shi et al. and initiated by the National Metabolic Management Center further identified a significant linear correlation between the TyG index and AF prevalence in diabetic populations (37). Meanwhile, a retrospective cohort study enrolling 409 patients with hypertrophic obstructive cardiomyopathy who underwent septal myectomy identified the TyG index as an independent risk factor for postoperative new-onset AF in this population, indicating its generalizability as a predictive biomarker for AF across diverse patient groups (30). A previous meta-analysis exploring the TyG index and AF reported that AF patients had higher TyG index levels than non-AF counterparts (SMD = 1.23, 95% CI: 0.71–1.75, *P* < 0.001) (36). Wang et al. performed a retrospective analysis of 2,242 AF patients admitted to two hospitals over an 18-month period and concluded that the TyG index may be independently correlated with AF recurrence after catheter ablation (25). In a follow-up study of AF patients receiving radiofrequency catheter ablation (RFCA), Yan et al. found that the cumulative TyG index measured within 3 months postoperatively could predict AF recurrence within one year, with an optimal cut-off value of 2.11 (31). In summary, as an effective surrogate marker reflecting insulin resistance (IR), the TyG index is promising to serve as a convenient biomarker for AF screening and prediction of new-onset AF. It is significantly positively correlated with AF risk and demonstrates independent predictive capacity in diabetic and non-diabetic individuals as well as patients following cardiac surgery.

**Figure 9:**
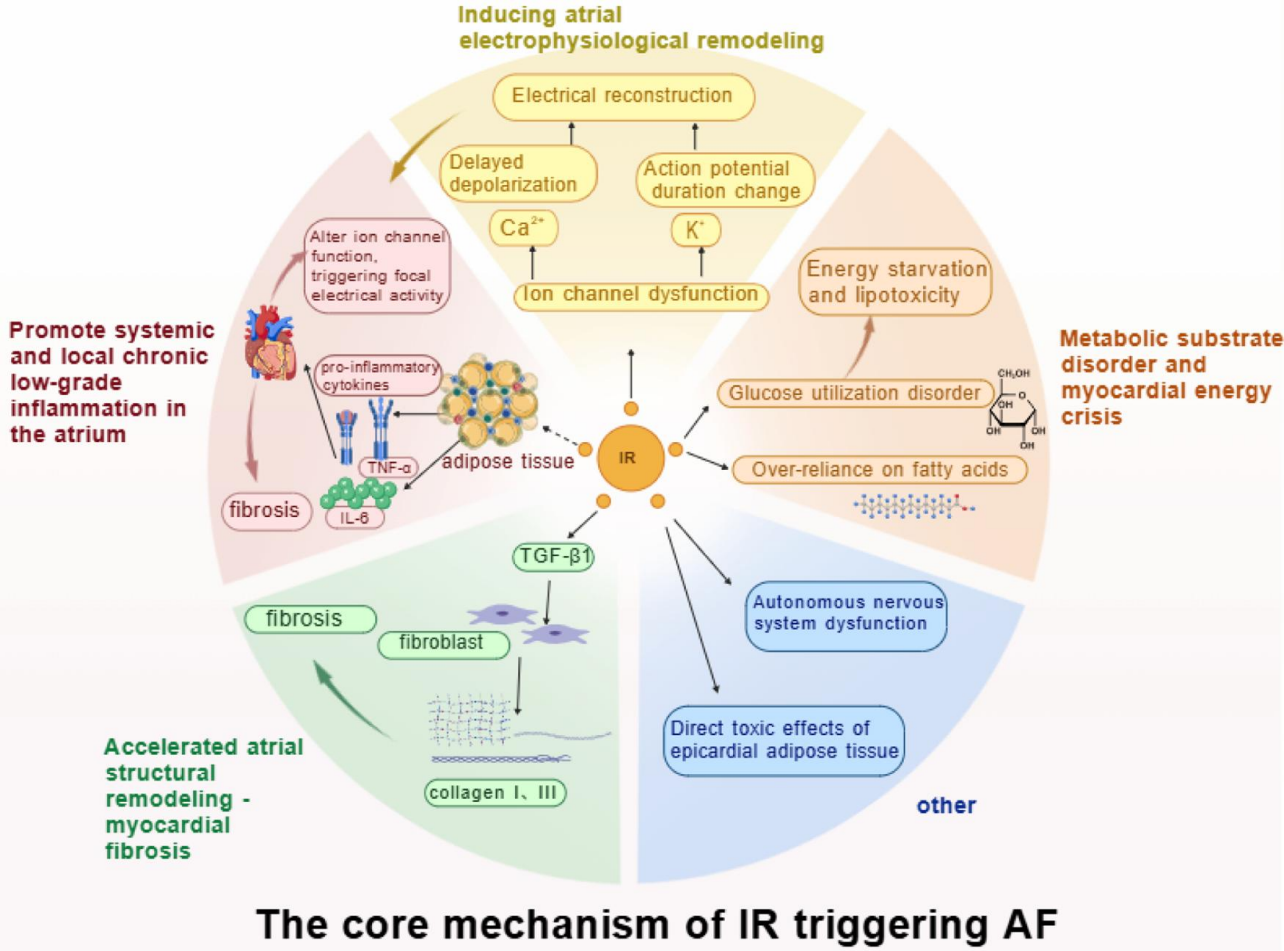
Proposed pathophysiological mechanism linking insulin resistance to increased atrial fibrillation incidence:the underlying cause for the higher incidence of atrial fibrillation (AF) in patients with an insulin resistance (IR) state lies in IR driving a self-reinforcing vicious cycle: metabolic dysregulation → inflammation & oxidative stress → structural & electrophysiological remodeling → arrhythmogenic substrate formation.

As a surrogate marker of insulin resistance (IR), the TyG index may be associated with AF via the following primary mediating pathways: 1. Atrial structural remodeling: IR activates the IRS-1/PI3 K/Akt and MAPK signaling cascades, facilitating the proliferation and phenotypic transformation of atrial fibroblasts as well as collagen deposition. These processes induce myocardial fibrosis and interstitial remodeling, which establish a structural substrate predisposing to AF initiation (38, 39). 2. Atrial electrophysiological remodeling: IR impairs the function of ion channels in cardiomyocytes, particularly L-type calcium channels and ryanodine receptors. This disruption disturbs intracellular calcium homeostasis, increases spontaneous calcium release, and provokes delayed afterdepolarizations, thereby triggering and sustaining AF (40). 3. Systemic inflammation and oxidative stress: IR is accompanied by low-grade chronic inflammation (elevated TNF-*α* and IL-6) and exacerbated oxidative stress, which injure atrial cardiomyocytes, accelerate fibrotic progression, and disrupt electrical conduction across atrial tissue (41). In addition, IR is linked to endothelial nitric oxide synthase (eNOS) uncoupling, which reduces nitric oxide (NO) bioavailability and induces endothelial dysfunction (11). 4. Autonomic nervous imbalance: IR and compensatory hyperinsulinemia activate the sympathetic nervous system and renin–angiotensin–aldosterone system (RAAS). The resulting autonomic imbalance acts as a critical mechanism underlying AF initiation and persistence (42). Collectively, these pathways form a vicious cycle: IR → metabolic dysregulation → inflammation/oxidative stress → atrial remodeling → arrhythmogenic substrate (See Figure 9 for details).

The clinical significance of this study lies in the potential of the TyG index to serve as a powerful tool for AF risk stratification and prevention, given its simplicity, low cost, and ease of implementation in primary healthcare settings. For individuals with an elevated TyG index, clinicians can identify them as a high-risk population for AF and implement early, targeted interventions. These may include intensive lifestyle management (e.g., Mediterranean diet, regular physical activity, weight control) and consideration of medications that improve insulin sensitivity (e.g., metformin). In AF patients who have undergone catheter ablation, a high TyG index may indicate a greater risk of recurrence, guiding the need for closer follow-up and more aggressive comprehensive management. Furthermore, integrating the TyG index with traditional AF risk prediction models (e.g., CHA₂DS₂-VASc score) could potentially enhance the predictive ability for AF and its complications (such as stroke), enabling more precise risk assessment.

### 4.1 Limitations

Several limitations of the present study should be acknowledged. First, all included studies adopted an observational design, which precludes the establishment of causal relationships between the TyG index and AF and leaves the possibility of reverse causality unaddressed. Residual confounding factors cannot be completely ruled out. Second, the total number of eligible studies was limited, with certain subgroups containing merely 2–3 studies, leading to insufficient statistical power to thoroughly assess all potential sources of heterogeneity. For instance, statin medication may directly lower the TyG index by reducing triglycerides and independently mitigate AF risk via anti-inflammatory effects, endothelial function improvement, and stabilization of atrial electrophysiology. However, few studies reported or adjusted for statin use, and relevant information was absent in most publications. Furthermore, multivariable meta-regression accounting for multiple covariates simultaneously was not feasible, so estimates of the independent contribution of each moderator may be distorted by residual confounding. Third, potential publication bias may have slightly overestimated the true magnitude of the association. Fourth, geographic and ethnic restrictions constitute a major drawback of this meta-analysis. Among the 19 included studies, 18 (94.7%) exclusively recruited Chinese participants, and only one multinational multicenter trial was included, which was still dominated by Asian populations. Such extreme geographic clustering substantially impairs the generalizability of our findings worldwide. Populations from distinct ethnicities and regions differ markedly in metabolic profiles (including baseline triglyceride and glucose concentrations and epidemiological patterns of IR), dietary habits (high-carbohydrate vs. high-fat dietary patterns), genetic backgrounds (divergent distribution of polymorphisms in metabolism-related genes such as TCF7L2 and PPARG), and risk factor profiles for AF. For example, well-documented disparities exist in triglyceride metabolism between East Asians and Western populations, and baseline TyG index levels and distributions differ substantially between Chinese and European/American cohorts. Accordingly, the effect sizes observed in this analysis (e.g., SMD = 0.65, OR = 2.14) cannot be directly generalized to non-Asian populations. Large external validation cohorts enrolling participants from Europe, North America, Africa, Latin America and other multiethnic groups are required in future research to verify whether the predictive performance of the TyG index for AF remains consistent across diverse ethnic and geographic groups. Fifth, uniform clinical cut-off values for the TyG index have not been established. Although our analysis confirmed a dose–response association between the TyG index and AF, no unified clinical warning threshold was identified. Reported cut-off points varied widely across individual studies, ranging from 8.23 to 9.57, and optimal thresholds may be modified by AF subtypes, population characteristics and ethnic backgrounds. This barrier hinders the translation of the TyG index from a research metric to a practical tool for clinical decision-making. Large-scale prospective trials utilizing receiver operating characteristic (ROC) curve analysis, Youden's index, net reclassification improvement (NRI) and other statistical approaches are warranted to determine optimal thresholds stratified by population and AF subtype. Sixth, this systematic review was not prospectively registered on PROSPERO or other dedicated registration platforms, representing a methodological shortcoming. Future analogous meta-analyses should register their protocols in advance in accordance with the PRISMA-P guidelines to improve research transparency and reproducibility.

## 5 Conclusions and future perspectives

In summary, based on currently available evidence predominantly derived from Asian populations (especially Chinese cohorts), the present meta-analysis preliminarily demonstrates that elevated triglyceride-glucose (TyG) index is strongly and significantly associated with increased risk of atrial fibrillation (AF) in this population. Such an association is consistently observed across multiple clinical scenarios, including new-onset AF, AF recurrence after catheter ablation, and postoperative AF. As a convenient and low-cost biomarker, the TyG index exhibits great potential for early AF screening, risk stratification, and the formulation of individualized prevention and treatment strategies.

Future research directions are proposed as follows: 1. Conduct large-scale, multicenter prospective cohort studies to further validate the predictive performance of the TyG index for AF and establish ethnicity-specific optimal cut-off values. 2. Design randomized controlled trials to investigate whether interventions reducing the TyG index can effectively lower the incidence or recurrence risk of AF, thereby providing more direct evidence to support a causal relationship. 3. Elucidate the molecular mechanisms underlying the link between the TyG index and AF, laying a theoretical foundation for the discovery of novel therapeutic targets. 4. Integrate the TyG index with emerging biomarkers, imaging parameters, and artificial intelligence models to construct more robust systems for AF prediction and prognostic evaluation.

## Supplementary material

The Supplementary Material for this article can be found online at: https://www.frontiersin.org/articles/10.3389/fcvm.2026.1818372 /full#supplementary-material
