# Supplementary material for: Triglyceride-glucose index and atrial fibrillation: a systematic review and meta-analysis
Source: Front Cardiovasc Med. 2026 Jul 29;13:1818372. doi: 10.3389/fcvm.2026.1818372 (PMC13463710; doi:10.3389/fcvm.2026.1818372)
Supplement: Supplementary file 1 [file Table1.pdf]

Appendix 1: Quality assessment results of the included studies (NOS)

Study	Author	Year	research design	select （4）	comparability （2）	Exposure/Outcomes （3）	total	quality grade
18	Li K	2025	Cross-section	3	1	2	6	medium-quality
19	Zuo Z	2025	Retrospective cohort	3	1	2	6	medium-quality
20	Song Y	2025	Cross-section	3	1	2	6	medium-quality
21	Wu X	2025	Retrospective cohort	3	1	2	6	medium-quality
22	Dong Z	2025	case-control	3	1	2	6	medium-quality
23	Chen S	2025	Retrospective cohort	3	1	2	6	medium-quality
24	Zhang Y	2023	Cross-section	3	1	2	6	medium-quality
25	Chen S	2022	case-control	3	1	2	6	medium-quality
26	Li X	2024	Retrospective cohort	3	2	2	7	high quality
27	Wang Z	2024	Retrospective cohort	3	2	2	7	high quality
28	Tang Q	2022	Retrospective cohort	3	2	2	7	high quality
29	Li T	2024	Retrospective cohort	3	1	2	6	medium-quality
30	He Y	2023	Retrospective cohort	3	1	2	6	medium-quality
31	Ling Y	2022	Retrospective cohort	3	2	2	7	high quality
32	Wei Z	2021	Retrospective cohort	3	2	2	7	high quality
33	Yan Q	2023	Retrospective cohort	3	2	2	7	high quality
34	Liu X	2023	Prospective Cohort	4	2	3	9	high quality
35	Li X	2025	Retrospective cohort	3	1	2	6	medium-quality
36	Zhao X	2025	Retrospective cohort	3	1	2	6	medium-quality

The NOS scores across the 19 studies ranged from 6 to 9, with 10 studies (52.6%) being high-quality (≥7 points), 9 studies (47.4%) being moderate-quality (5–6 points), and no low-quality studies identified. The only study achieving a full score (9 points) was Liu X's 2023 study, which is the sole prospective cohort design. All cross-sectional and case-control studies received a score of 6; the primary reasons for lower scores were: (1) inability to establish a temporal relationship between exposure and outcome; (2) insufficient representativeness in selecting non-exposure groups or control groups. Although overall quality was acceptable, the very low proportion of prospective studies (5.3%) limited the strength of
