# Supplementary material for: Triglyceride-glucose index and atrial fibrillation: a systematic review and meta-analysis
Source: Front Cardiovasc Med. 2026 Jul 29;13:1818372. doi: 10.3389/fcvm.2026.1818372 (PMC13463710; doi:10.3389/fcvm.2026.1818372)
Supplement: Supplementary file 2 [file Table2.pdf]

Appendix 2: Fully adjusted multivariate model

Study	Author	Year	research design	multitude	AF type	Multivariate adjustment for covariates	Unadjusted important confounding factors
18	Li K	2025	Cross-section	Hypertensive	New	Age, gender, BMI, systolic blood pressure, diastolic blood pressure, heart rate, fasting blood glucose, LDL-C, HDL-C, serum uric acid, creatinine	Disease duration of diabetes, types of antihypertensive medications, sleep apnea, alcohol consumption
19	Zuo Z	2025	Retrospective cohort	Sepsis	New	Age, gender, BMI, SOFA score, APACHE II score, stress hyperglycemia ratio, white blood cell count, procalcitonin, lactate level, mechanical ventilation, vasoactive drugs	History of AF, electrolyte disturbances, source of sepsis, type of antibiotics
20	Song Y	2025	Cross-section	CAD	New	Age, gender, BMI, smoking status, hypertension, diabetes mellitus, LDL-C, HDL-C, TG, TC, hs-CRP, Gensini score	Cardiac function classification, medication use (statins/antiplatelet agents), alcohol consumption
21	Wu X	2025	Retrospective cohort	AMI	New	Age, gender, BMI, Killip classification, LVEF, number of stents, infarction-related arteries, fasting blood glucose, LDL-C, TC	Preoperative medication, postoperative medication, timing of revascularization, renal function
22	Dong Z	2025	case-control	Subjects	New	Age, gender, BMI, hypertension, diabetes mellitus, coronary heart disease, stroke, smoking, alcohol consumption	Exercise habits, dietary patterns, sleep apnea, thyroid function
23	Chen S	2025	Retrospective cohort	AMI	New	Age, gender, BMI, Killip classification, LVEF, TC, TG, LDL-C, HDL-C, fasting blood glucose	Cardiac function classification, medication use, renal function, inflammatory markers
24	Zhang Y	2023	Cross-section	NAFLD	New	Age, gender, BMI, waist circumference, hypertension, diabetes mellitus, ALT, AST, GGT, platelet count, FIB-4 index	Alcohol consumption volume, liver biopsy results, detailed scoring of metabolic syndrome components
25	Chen S	2022	case-control	Non-diabetic	New	Age, gender, BMI, systolic blood pressure, diastolic blood pressure,TC, TG, LDL-C,HDL-C, fasting blood glucose, and serum uric acid	Exercise habits, dietary patterns, insulin levels, HOMA-IR
26	Li X	2024	Retrospective cohort	RFCA	palindromia	Age, gender, BMI, AF type, AF duration, left atrial diameter, LVEF, CHA_2DS_2-VASc score, HAS-BLED score, intraoperative ablation strategy	Postoperative medications (antiarrhythmic agents/anticoagulants), postoperative inflammatory markers, and postoperative lifestyle
27	Wang Z	2024	Retrospective cohort	RFCA	palindromia	Age, gender, BMI, AF type, left atrial diameter, LVEF, NT-proBNP, hs-CRP, postoperative medications	Preoperative medication, details of ablation strategy, and postoperative follow-up frequency
28	Tang Q	2022	Retrospective cohort	RFCA	palindromia	Age, gender, BMI, AF type, AF duration, left atrial diameter, LVEF, postoperative medications	Preoperative metabolic status, postoperative lifestyle, genetic factors
29	Li T	2024	Retrospective cohort	RFCA	palindromia	Age, gender, BMI, AF type, left atrial diameter, LVEF, postoperative medications	Preoperative medication, ablation strategy, postoperative inflammation
30	He Y	2023	Retrospective cohort	RFCA	palindromia	Age, gender, BMI, AF type, AF duration, left atrial diameter, LVEF, postoperative medications	Preoperative metabolic indicators and postoperative lifestyle
31	Ling Y	2022	Retrospective cohort	STEMI-PCI	POAF	Age, gender, BMI, Killip classification, LVEF, infarction site, number of stents, fasting blood glucose, TC, TG	Preoperative medication, postoperative medication, timing of revascularization, renal function
32	Wei Z	2021	Retrospective cohort	HOCM	POAF	Age, gender, BMI, NYHA classification, left ventricular outflow tract pressure gradient, SAM sign, surgical approach, postoperative medications	Preoperative medication, intraoperative procedures, and postoperative management
33	Yan Q	2023	Retrospective cohort	RFCA	palindromia	Age, gender, BMI, AF type, AF duration, left atrial diameter, LVEF, postoperative medications, cumulative TyG index	Single preoperative TyG injection, postoperative lifestyle, and genetic factors
34	Liu X	2023	Prospective Cohort	without known CVD	New	Age, gender, BMI, systolic blood pressure, diastolic blood pressure, TC, TG, LDL-C, HDL-C, fasting blood glucose, serum uric acid, smoking, alcohol consumption, physical exercise	Detailed evaluation of thyroid function, sleep apnea, and dietary patterns
35	Li X	2025	Retrospective cohort	RFCA	palindromia	Age, gender, BMI, AF type, left atrial diameter, LVEF, postoperative medications, coronary artery fat attenuation index	Preoperative metabolic status and postoperative lifestyle
36	Zhao X	2025	Retrospective cohort	HFpEF+ RFCA	palindromia	Age, gender, BMI, AF type, left atrial diameter, LVEF, NT-proBNP, postoperative medications, severity of HFpEF	Preoperative medication, ablation strategy, and postoperative management
